# Recent Advances in Functionalized Nanoparticles in Cancer Theranostics

**DOI:** 10.3390/nano12162826

**Published:** 2022-08-17

**Authors:** Sarkar Siddique, James C. L. Chow

**Affiliations:** 1 Department of Physics, Toronto Metropolitan University, Toronto, ON M5B 2K3, Canada; 2 Radiation Medicine Program, Princess Margaret Cancer Centre, University Health Network, Toronto, ON M5G 1X6, Canada; 3 Department of Radiation Oncology, University of Toronto, Toronto, ON M5T 1P5, Canada

**Keywords:** functionalized nanoparticles, MRI-guided therapy, molecular imaging, biomedical imaging, cancer therapy, cancer theranostics

## Abstract

Cancer theranostics is the combination of diagnosis and therapeutic approaches for cancer, which is essential in personalized cancer treatment. The aims of the theranostics application of nanoparticles in cancer detection and therapy are to reduce delays in treatment and hence improve patient care. Recently, it has been found that the functionalization of nanoparticles can improve the efficiency, performance, specificity and sensitivity of the structure, and increase stability in the body and acidic environment. Moreover, functionalized nanoparticles have been found to possess a remarkable theranostic ability and have revolutionized cancer treatment. Each cancer treatment modality, such as MRI-guided gene therapy, MRI-guided thermal therapy, magnetic hyperthermia treatment, MRI-guided chemotherapy, immunotherapy, photothermal and photodynamic therapy, has its strengths and weaknesses, and combining modalities allows for a better platform for improved cancer control. This is why cancer theranostics have been investigated thoroughly in recent years and enabled by functionalized nanoparticles. In this topical review, we look at the recent advances in cancer theranostics using functionalized nanoparticles. Through understanding and updating the development of nanoparticle-based cancer theranostics, we find out the future challenges and perspectives in this novel type of cancer treatment.

## 1. Introduction

**Figure 1:**
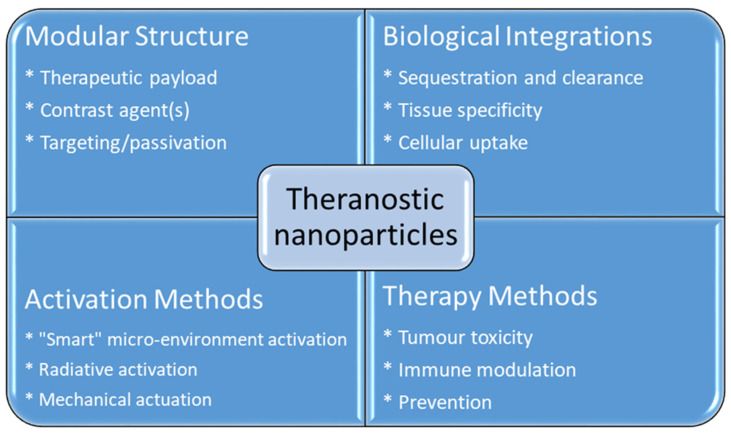
Advantages using nanoparticles in cancer theranostics.

Cancer treatment has gained considerable attention in biomedical research over the past few decades due to the serious threat it poses to human health. The mortality rate of cancer increases every year, which leads to the need for the development of more efficient cancer therapeutic strategies [1]. Even though there is a major advance in cancer therapy, it continues to be a significant challenge due to tolerability and adherence [2]. Theranostics is a term first used by John Funkhouser at the beginning of the 1990s. It is defined as a combination of diagnostic tools that are the most suitable for specific diseases [3]. Theranostics portrays a close connection between diagnostics and the consequent therapy, and the theranostic principle has attracted huge attention in personalized medicine, in particular oncology. This allowed tumours at the advanced stage to be treated accurately with fewer side effects. For decades theranostics have been used for the therapy of benign and malignant thyroid diseases; however, recently, theranostics have been applied to other malignancies [4]. Theranostics agents such as radioisotopes, liposomes, quantum dots and plasmonic nanobubbles can be attached to anticancer drugs, imaging agents and cancer cell markers with the support of imaging techniques, providing the potential to facilitate the diagnosis, treatment and management of cancer patients [5]. The development of highly sensitive imaging modalities such as SPECT and PET with the synthesis of novel radiolabelled molecules specific for different biochemical targets promoted nuclear medicine into a new era [6]. These molecular imaging modalities have been applied in cardiology, neuroscience, oncology, gene therapy and theranostics. Nanoparticles (NPs) have been used as therapeutic or imaging agents that enhance the efficacy and control biodistribution and reduce the toxicity of drugs. In 2014–2015, there were 51 FDA-approved nanomedicines that met the definition of nanomedicines as therapeutic or imaging agents, and 77 products in clinical trial [7]. One of the crucial characteristics of nanomaterials is their small size. Their high affinity, high specificity, high thermal stability, low off-target accumulation and good solubility are among many adventurous characteristics they possess in cancer therapy. They can penetrate dense tissues of the tumour very well [8]. Nanotechnology in medicine is currently developed for drug delivery, and many substances are under study for cancer therapy. Solid NPs can be used for drug targeting when they reach the intended diseased site in the body, and the toxicology of the drug nanocarriers has been evaluated [9]. Active targeting is accomplished by conjugating tumour-specific ligands to the NPs’ surface. It complements the enhanced permeability and retention effect (EPR). EPR is a universal pathophysiological phenomenon and mechanism where macromolecules with certain sizes above 40 kDa can progressively accumulate in the tumour vascularized area and achieve targeted delivery and retention of the anticancer compound into the solid tumour [10]. Some of the particles that are used to functionalize NPs are antibodies or antibody fragments, human transferrin protein, peptides, carbohydrates and vitamins. These biomarkers are recognized by their representative targeting ligands such as epidermal growth factor, human epidermal growth factor 2, Mucin-1, nucleolin, epithelial cell adhesion molecule and platelet-derived growth factor receptor 2. For anticancer drug delivery, Fu et al. [11] proposed to use aptamer-functionalized nanoparticles. This is because aptamers have favourable features such as a small size, very low immunogenicity, low cost of production and high affinity and specificity. The advantage of NPs as a theranostics agent is shown below in Figure 1 [12].

A study of the functionalized NPs by wrapping them in the cancer cell membrane showed that the resulting particle possesses an antigenic exterior closely resembling that of the source cancer cells. These NPs allowed immunological adjuvant and membrane-bound tumour-associated antigens to be efficiently delivered to the cancer cell and promote an anticancer immune response [13]. Mesoporous silica NPs have a high potential in theranostic applications. They have a wide array of formulations and have significant in vivo efficacy for treating myriad malignant diseases in preclinical models [14]. The treatment of oral cancer is difficult and has a poor survival rate. Studies show that the proper inhibition of GST by NPs is promising in reversing pingyangmycin and carboplatin drug resistance in oral cancer, which improves the treatment outcome significantly [15]. One of the issues to keep in mind when using NPs is the formation of oxidative stress, which can have life-threatening consequences [16].

As there are many advantages of using functionalized nanoparticles in cancer theranostics, and there are various studies that have been carried out and are in progress, organizing and reviewing the recent works are necessary to see the big picture. From the current contributions in different aspects, we will be able to find out the future trends of work.

## 2. Magnetic Resonance Imaging (MRI)

**Figure 2:**
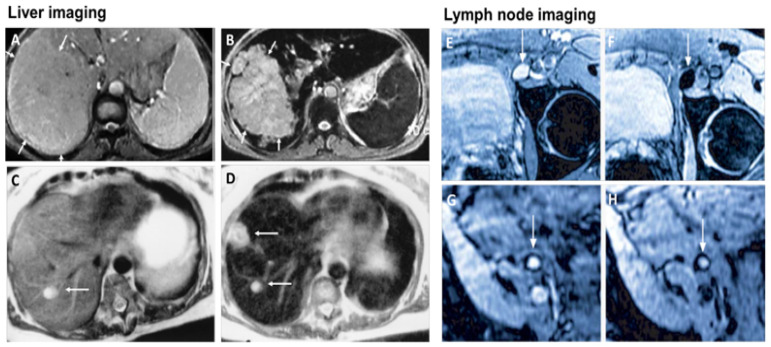
Superparamagnetic iron oxide NPs being used in liver imaging and lymph node imaging. (**A**,**B**): T2-weighted MR image of a liver with a large hepatocellular carcinoma before (**A**) and after (**B**) the administration of SPION. The lesion is demarcated with arrows. (**C**,**D**): Standard (**C**) and SPION-based contrast-enhanced (**D**) MR imaging of liver metastasis in a patient with colorectal cancer. After administration of ferumoxide SPION, a second metastasis becomes visible on T2-weighted MR image. (**E**,**H**): Lymph node in left iliac region (arrow), with and without metastatic infiltration. T2-weighted images before (**E**,**G**) and 24 h after (**F**,**H**) administration of ferumoxtran. Lymph node (arrow) appears bright before injection of UPIO (**E**,**G**). One day after injection, a signal loss in the lymph node (arrow) due to high UPIO macrophage uptake can be observed, thus indicating functionality and no metastasis (**F**). Conversely, in the lower panel, the lymph node (arrow) stays bright, indicating no trafficking of USPIO and thus metastatic colonization (**H**). Reprinted with permission from Ref. [30]. Copyright 2020 Elsevier.

MRI is one of the most powerful means of clinical detection and prognosis observation [17]. MRI is an imaging modality that is non-invasive, and it provides comprehensive multi-parametric information generally used for brain imaging [18]. MRI benefits from the contrast agent that provides a more improved depiction of large and medium-sized vassals and can provide dynamic vascular/perfusional properties of tissues. Gadolinium (Gd)-based contrast agents are widely used in MRI [19,20]. MRI can be coupled with other therapy to provide image-guided therapy for better treatment outcomes and tumour-targeting ability [21]. A study synthesized a multifunctional Gd-DTPA-ONB lipid by adding the Gd-DTPA contrast agent to an o-nitro-benzyl ester lipid. It combines the MRI tracking ability with dual trigger release capabilities, which allow maximum sensitivity without reducing the drug encapsulation rate. It can be activated by both PH-trigger hydrolysis and photo treatment [22]. Another Gd nanocomposite was synthesized by decorating Gd NPs onto the graphene oxide, and then functionalized with polyethylene glycol and folic acid. It was used to load doxorubicin to accomplish targeted image-guided drug delivery with MRI [23]. Liposomes are a useful class of NPs due to their tunable properties and multiple liposomal drug formulation. They have been clinically approved for cancer treatment. A vast number of Gd-based liposomal MRI contrast agents have been developed that can be used for targeted image-guided drug delivery [24]. Chemical exchange saturation transfer MRI has important advantages such as its ability to detect diamagnetic compounds that are not detectable using conventional MRI. It makes a broad spectrum of bioorganic agents, nanocarriers and natural compounds directly MRI detectable with a high resolution. It is advantageous for image-guided drug delivery [25]. An in vivo study looked at amphiphilic polymer-coated magnetic iron oxide NPs that were conjugated with near-infrared (NIR) dye-labelled HER2 affibody and chemotherapy drugs. Cisplatin was the drug used as the chemotherapy drug. MRI-guided therapy and the optical imaging detection of the therapy-resistant tumour were examined in an orthotopic human ovarian cancer xenograft model with a high level of HER2 expression. The result shows it significant inhibited the primary tumour and peritoneal and lung metastases in the ovarian cancer model in mice [26]. Another study looked at the NP with a unique morphology, which consists of a superparamagnetic iron oxide core and star-shaped plasmonic shell with high aspect ratio branches. Its strong near-infrared responsive plasmonic properties and magnetic properties allow it to be used in multimodal quantitative imaging, which combines the advantageous functions of MRI, magnetic particle imaging (MPI) and photoacoustic imaging. It can be used for image-guided drug delivery with tunable drug release capacity [27]. Drug resistance in chemotherapy has been a challenge for a long time in pancreatic cancer due to the stomal barrier making it difficult to reach the tumour microenvironment. A study developed IGF1 receptor-directed multifunctional theragnostic NPs for the targeted delivery of Dos into IGF1R-expressing drug-resistant tumour cells and tumour-associated stromal cells. NPs were prepared by combining IGF1 with magnetic iron oxide NPs carrying dox. They provided an excellent theranostics platform and showed good tumour control in an in vivo study [28]. Superparamagnetic iron oxide NPs have also been widely used in MRI and nanotheranostics. They can be coated with a biocompatible polymer such as polyethylene glycol or dextran, which allows chemical conjugation. They have a very high potential in MRI-guided drug delivery [29]. Figure 2 shows superparamagnetic iron oxide NPs being used in liver imaging and lymph node imaging [30].

### 2.1. MRI-Guided NPs for Gene Therapy

Gene therapy has gained considerable attention over the years and the health community has gained much more new information and knowledge regarding gene therapy [31]. Gene therapy is a form of engineered viruses carrying a therapeutic agent or containing genetically modified cells such as when chimeric antigen receptors are introduced to the T lymphocytes for cancer therapy such as for leukemia [32]. New gene therapy has shown its potential to significantly improve the survival rate of cancer patients [33]. For cancer gene therapy, the therapeutic agent generally requires a carrier such as an NP. MRI allows the tracking of that carrier and allows image-guided therapy, which can significantly improve the outcome [34]. A study looked at low molecular weight poly (ethylenimine)-poly (ethylene glycol) nanogels loaded with transforming growth factor -β1 siRNA and ultra-small iron oxide NPs for gene therapy and a T1-weighted MRI of tumour and tumour metastasis in a mouse sarcoma model. The study result shows it enhances the MRI image and effectively delivers the siRNA and inhibits tumour growth in the subcutaneous sarcoma tumour model and lung metastasis by silencing the TGF-β1 gene [35]. Another study investigated shaped, controlled magnetic mesoporous silica NPs and their performances in magnetic resonance image-guided targeted hyperthermia-enhanced suicide gene therapy of hepatocellular carcinoma. They had a higher loading capacity and better magnetic hyperthermia properties. They also had decreased cytotoxicity [36]. A bowl-shaped Fe_3_O_4_ NP with a self-assembly concept and appropriately surface-functionalized was studied with the aim for it to be used as a multifunctional carrier in combination therapy and gene therapy. The in vivo result shows promising results in the mouse breast cancer model [37]. The catalytic deoxy ribozyme has great potential in gene therapy via gene regulation but requires the carrier to reach the tumour target. A study showed polydopamine-Mn^2+^ NPs to be effective carriers and together they can be used as a photothermal agent and contrast agent for photoacoustic and magnetic resonance imaging [38]. Another study developed Fe_3_O_4_@PDA NPs to transport siRNA for gene therapy. The NPs were coated with mesenchymal stem cells to form a membrane. The overall complex showed good transport ability and photothermal functionality, and enhanced MRI capability [39].

### 2.2. MRI-Guided NPs for Thermal Therapy

**Figure 3:**
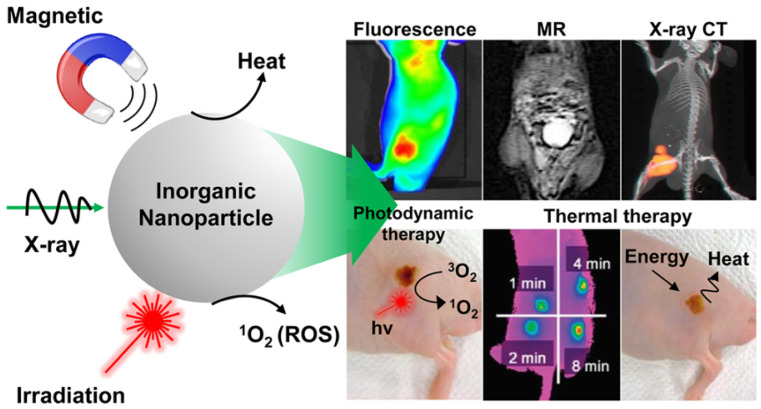
Applications of inorganic NPs for cancer therapy and imaging. Reproduced with permission from [40]. Copyright 2017 ACS Publications.

Light-activated therapies have been introduced for cancer treatment for numerous cancers. Two of the main methods are localizing chemical exchange on the tumour known as photodynamic therapy (PDT) and localized thermal damage to the tumour, also known as photothermal therapy (PTT) [40]. Inorganic NPs have gained significant attention in image-guided thermal therapy in recent years, and the applications of inorganic NPs in tumour imaging and therapy are shown in Figure 3. The NPs contain metal, a semiconductor, metal oxide, nanocrystal and lanthanide-doped up conversion NPs. They can generate heat and reactive oxygen species, so they are ideal for image-guided PTT [41]. The thermal energy also promotes the gasification of perfluoropentane to enable the visualization of cancer tissue in ultrasound imaging, as well as enhances MRI imaging, and makes it ideal for dual MRI ultrasound imaging [42]. Core/shell nanoparticles were investigated for MRI imaging, magnetic hyperthermia and PTT due to their surface being coated with a porous shell. It can entrap large quantities of water around the nanoparticles and allows enhanced and efficient water exchange, which provides an improved magnetic resonance contrast signal. It also helps with NIR absorbance of the core and can have an enhanced thermal effect via synergistic PTT and magnetic hyperthermia. The nanoparticles investigated for this purpose were MnFe_2_O_4_/PB [43]. Another study developed temperature-activated engineered neutrophils by combining indocyanine green-loaded magnetic silica NIR sensitive nanoparticles. It provides a platform for dual-targeted PTT. The combination of magnetic targeting and neutrophil targeting provides an enhanced accumulation of the photothermal agent at the tumour site [44]. A study wrapped together gadolinium-DTPA, indocyanine green and perfluoropentane in a poly (lactic-co-glycolic) acid shell membrane by a double emulsion approach. Under NIR the indocyanine green converts the optic energy into thermal energy and converts oxygen to singlet oxygen, which destroys cancer cells through PTT and PDT. Another nanotheranostics agent was prepared via the participation of hydrophilic CuS nanoparticles, styrene, methacrylic acid, N-isopropylacrylamide and a polymerizable rare earth complex. It had good biocompatibility with a high loading capacity for DOX-HCI. Drug release can be activated via PH or high temperature. All these properties make it ideal for PTT and chemotherapy. MRI can also be used on it for image-guided drug delivery [45]. CuS material shows poor MRI ability but excellent photo absorption ability, whereas Fe-based materials have good MRI ability. A study combined the two and made a Cu_x_Fe_y_S_z_ sample that includes CuFeS_2,_ FeS_2_ and Cu_5_FeS_4_ nanomaterials. The study result shows it to have high potential in MRI-guided photothermal enhanced chemo dynamic therapy [46].

### 2.3. Magnetic Hyperthermia Treatment (MHT)

In cancer treatment, the use of a magnetic implant as a thermal seed exposed to the alternating magnetic field is the primary principle behind magnetic hypothermia. Magnetic hypothermia has been used for cancer treatment since the 1950s [47]. Traditionally, deep tumour treatment via magnetic fluid hyperthermia was not possible due to the very low-frequency excitation field being no longer than 100 m in vivo. Now it is possible due to NPs and magnetic particle imaging [48]. In magnetic hyperthermia, the tumour is heated to a moderate temperature of 40–30 °C to destroy cancer cells without the side effects associated with conventional treatment. It can also be co-administered with conventional treatment for better outcomes [49]. Iron oxide NPs have been employed as intra-tumour MTH agents in brain and prostate tumour clinical trials [50]. A study looked at encapsulating produced magnetic iron oxide nanocomposites due to their excellent magnetic saturation and superior magnetic to thermal conversion efficiency with a specific absorption range. It shows the good potential for magnetic hyperthermia therapy [51]. A side effect of magnetic hypothermia is heating of the tumour’s surrounding tissue, which is aimed to be minimized as much as possible [52]. Using NPs can localize the heat and minimizes the damage to the tissue. One example is the release of heat due to the transfer of magnetic field energy into heat by adding magnetic NPs to the tumour in a time-varying magnetic field. This heats the cancer cells, whereas surrounding non-malignant tissues can be spared [53].

### 2.4. MRI-Guided Chemotherapy

**Figure 4:**
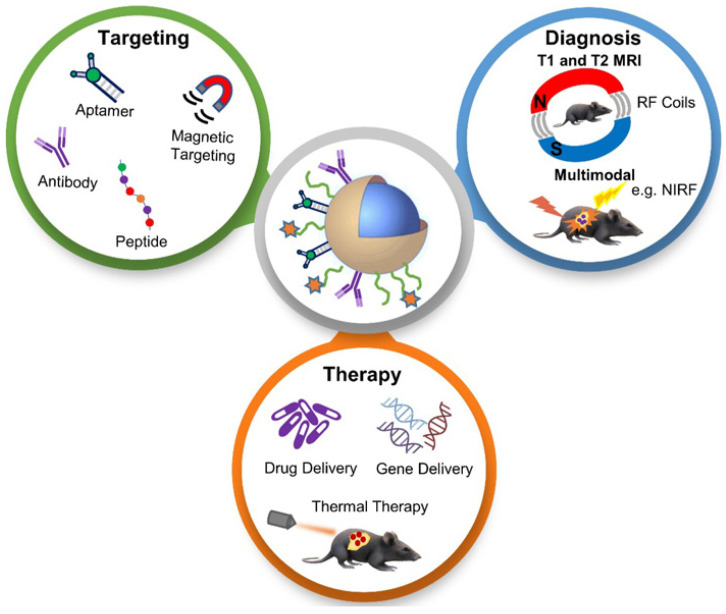
Schematic diagram showing applications of functionalized magnetic NPs in MRI-based diagnosis and anticancer therapy. Reproduced with permission from [55]. Copyright Anani et al. 2020.

NPs with magnetite composition and polymer encapsulation are used in many applications as theranostic agents for drug delivery and MRI [54]. MRI provides a high-resolution image of structures in the body, and when combined with other imaging modalities, together they can provide complementary diagnostic information for more accurate tumour characteristics identification and the precise guidance of anticancer therapy [55]. The applications of functionalized magnetic NPs in cancer nanotheranostics are shown in Figure 4. Magnetic NPs can be functionalized and guided by a magnetic field. They allow advanced MRI-guided gene and drug delivery, magnetic hyperthermia cancer therapy, cell tracking and bioseparation and tissue engineering [56]. Iron oxide NPs can be used in the diagnosis of liver, inflammation and liver and vascular imaging via MRI. They are also used for therapeutic applications such as iron supplementation in anaemia, macrophage polarization, magnetic drug targeting and magnetic fluid hyperthermia. Due to these properties, they are very useful in theranostic applications [30]. A multifunctional theranostic platform was developed based on amphiphilic hyaluronan/poly-(N-ε-Carbobenzyloxy-L-lysine) derivative (HA-g-PZLL) superparamagnetic iron oxide and aggregation-induced emission (AIR) NPs for magnetic resonance and fluorescence dual-modal image-guided PDT [57]. Gadolinium-based NPs have high relaxivity, passive uptake in the tumour due to an enhanced permeability and retention effect, and adapted biodistribution. These properties make them ideal contrast agents for positive MRI imaging. They can also act as an effective radiosensitizer in radiotherapy, neutron therapy and hadron therapy [58]. Ultra-small gold NPs have low toxicity, and they are non-immunogenic by nature. They have fast kidney clearance and can be used in NIR resonant biomedical imaging modalities. They can be used as an enhancer in MRI, photoacoustic imaging, X-ray and fluorescence imaging. They can also be used to generate heat and local hyperthermia of cancer tissue in PTT. They can also be functionalized to deliver the drug to the cancer cells. All these properties make them ideal for theranostic applications [59]. Another study synthesized a polydopamine-coated manganese oxide NP (FA-Mn_3_O_4_@PDA@PEG) conjugate for MRI-guided chemo (PTT). It has a relaxivity of 14.47 mM^−1^ s^−1^, which makes it an excellent contrast agent for MRI [60].

## 3. Immunotherapy

Cancer immunotherapy aims to improve the antitumour immune response, which has advantages over chemotherapy such as fewer off-target effects [61]. T-cell checkpoint inhibitors are crucial in the management of advanced cancers such as melanoma and non-small cell lung cancer [62]. Immunotherapy needs to be personalized because of the variance in the immune response from patient to patient. Cancer immunotherapy includes pharmaceuticals such as monoclonal antibodies, immune checkpoints, cell therapy and vaccines. Programmed cell death is achieved in a combination of program cell death protein 1 and programmed cell death protein ligand 1 drugs and other immune therapy drugs such as antibody–drug conjugates, and other therapies such as chemotherapy and radiation therapy [63]. Immunotherapy can also be conjugated with positron emission tomography and single-photon emission computed tomography to evaluate the response to immune checkpoint therapy [64]. Nano immunotherapy has three different mechanisms, targeting cancer cells, targeting the peripheral immune system and targeting the tumour microenvironment. When it is targeting the cancer cells, it aims to promote immunogenic cell death by releasing the tumour antigens. When it is targeting the microenvironment, it inhibits immunosuppressive cells such as M2-like tumour-associated macrophages. It also reduces the expression of immunosuppressive molecules, e.g., changing growth factor beta. When it is targeting the peripheral immune system, it aims to promote T cell production in secondary lymphoid organs, and also engineer and strengthen the peripheral effector immune cell population, which ultimately promotes anticancer immunity [65]. Liposomal NPs have a very high potential to deliver immune modulators and act as theranostic agents [66]. NPs of different types such as graphene oxide, black phosphorous, silver, gold, copper, tellurium, iron oxide, zinc oxide and magnesium oxide, prepared using the aerosol method, have many advantages and show high potential in cancer theranostics [67]. Wrapping the NPs with a cellular membrane shows a high potential for cancer theranostics. They are generally isolated from immune cells, stem cells, blood cells and cancer cells and allow for superior tumour targeting through self-recognition, homotypic targeting and prolonged systemic circulation [68]. Magnetic NPs as novel agents for cancer theranostic purposes play a big role in treating malignant melanomas and significantly improves the treatment outcome [69].

## 4. Photothermal Therapy (PTT) and Photodynamic Therapy (PDT)

**Figure 5:**
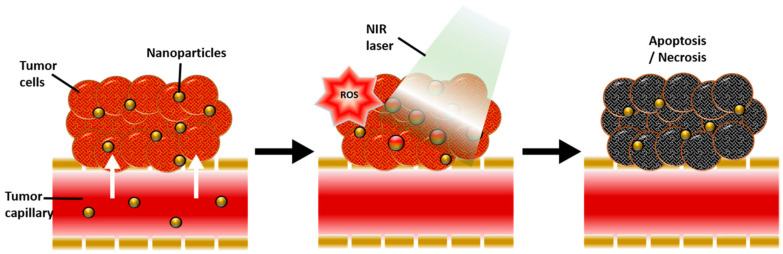
Schematic diagram showing the physiological and biological effects of gold nanoparticle-mediated photothermal therapy (PTT) and photodynamic therapy (PDT). A large amount of gold nanoparticles accumulate due to the leaky vasculature of the tumour, resulting in a photothermal effect in response to near-infrared (NIR) light and reactive oxygen species (ROS) generated by secondary delivered photosensitizer (PS), ultimately inducing apoptosis and necrosis of tumour tissue. Reproduced with permission from [74]. Copyright Kim et al. 2018.

Research on gold NPs has increased significantly in recent years due to their property advantages and theragnostic compatibilities. They have been widely used in cancer theragnostics including photo imaging and PTT due to their stability, enhanced solubility, bifunctionality, biocompatibility and cancer-targeting ability [70]. A study functionalized AuNP with hyaluronic acid, polyethylene glycol and adipic dihydrazide. The antitumour drug was loaded into the NPs via the chemical method. The result shows the NPs had very low toxicity toward cells in high doses with a significant enhancement of the antitumour properties [71]. PTT therapy has high compatibility to be combined with other therapies to yield better treatment outcomes. One of the limitations of PPT is its light penetration depth that can cause the incomplete elimination of cancer cells, which could lead to tumour recurrence and metastases in distant organs. This shortcoming can be eliminated by combining PTT with other therapies [72]. Glioblastoma multiforme therapeutic efficacy is often limited due to the poor penetration of therapeutics through the blood–brain barrier. Functionalized up conversion of an NP-based delivery system can target brain tumours and convert deep tissue penetrating NIR light into visible light for PPT and PDT [73]. In PPT and PDT, the heat generation and the activation of photosensitizer drugs occurs in response to exogenously applied light of a specific wavelength. The NPs allow the generation of cytotoxic photothermal heating via a surface plasmon resonance phenomenon and reactive oxygen species. This cytotoxic heat promotes apoptotic and necrotic cancer cell death. Gold NPs can be used both as photothermal agents and photosensitize carriers due to their surface plasmon resonance effect that has a very high efficiency of light to hear conversion and simple thiolation chemistry for functionalization, which allows targeting [74]. The mechanism of the photothermal and photodynamic therapy using gold NPs can be seen in Figure 5 using near-infrared light. A study also looked at conjugating curcumin to the gold NPs to be used in PTT. Curcumin is a polyphenol with an anticancer and antimicrobial ability, and gold NPs allow it to be transported to the target site [75]. Gold NPs have proved themselves to be an excellent theranostic agent for carrier and synergistic PTT and PDT due to their properties [74]. PEGylated bovine serum albumin-coated silver core/shell NPs were proposed for PPT due to their advantageous properties and ability to transport indocyanine green, a clinically-approved NIR dye. The study shows it is an effective carrier and an efficient agent in PPT [76]. A study used magnetite (Fe_3_O_4_) NPs that were functionalized with chlorin e6 and folic acid as a theragnostic agent in PDT and showed that it can be used as a versatile therapeutic tool that can be used in diagnostic imaging [77]. A study synthesized novel carbon dots/hemin NPs. The fluorescence resonance energy transfer effect enhances their photothermal ability and synergises with PDT [78]. Another study synthesized selenide molybdenum nanoflower that is capable of delivering NIR-mediated synergetic PTT and PDT [79]. A cost-effective modified zinc oxide NP was also introduced that has NIR absorbance, which can be used in PTT and PDT for synergistic therapy [80]. A study also looked at gold doped hollow mesoporous organosilica NPs for PDT and PTT with multimodal imaging for gastric cancer [81]. These functionalized NPs have been suggested for non-invasive cancer treatment because the near-infrared-induced PTT and PDT effect can increase the cancer cell kills.

## 5. Molecular Imaging

A nanoscaled material’s size, shape surface chemistry and structure allow their functionalization and utilization in theranostic applications [82]. Molecular imaging shows their high potential in the identification of inflammatory cellular and molecular processes in cardiovascular disease. NPs have been studied as contrast agents in molecular imaging in the detection of vascular inflammation [83]. Quantum dot has also shown very good results in an in vivo study of molecular imaging as a contrast agent [84]. A preclinical study showed that molecular ultrasound imaging has high sensitivity and specificity in disease detection, classification and therapy response monitoring. The use of microbubbles may have high potential in cancer detection [85]. Perfluorocarbon NPs have a high potential to be used in combination with imaging modalities for targeted drug delivery. Their intravascular constraint from their particle size provides a unique advantage for angiogenesis imaging and antiangiogenesis therapy [86]. Gold NPs have been extensively used as a contrast agent in molecular imaging and as a theranostics platform [87]. Silica NPs have also been used in molecular imaging and as a theranostic platform due to their having different sizes in nanometer ranges, and this allows surface modification. It also allows conjugation of different biomolecules such as nucleic acid and proteins [88].

## 6. Chemotherapy

Some of the common issues with old therapeutic agents are their poor water solubility, non-specific distribution and lack of targeting capabilities. Now, functionalized NPs overcome those shortcomings and can also act as a contrast agent for diagnosis in therapeutic applications [89]. Therapeutic NPs can efficiently deliver chemotherapeutic drugs to the pathological site. This avoids accumulation in healthy organs and tissue and is based on an enhanced permeability and retention effect [90]. NPs offer several advantages in that they are drug-like, their capability to carry high payloads of a drug with reduced toxicity of the drug and prolonged half-life, and, most importantly, their increased targeting efficiency. All of these capabilities make them excellent theranostic agents and allow theranostic applications to flourish [91]. NPs are captured and eliminated by the natural immune system and this is an inconvenience for drug delivery. Camouflaging NPs with cell membrane provides a solution to this obstacle. A novel class of NPs such as biomimetic NPs was developed, which can inherit specific biological functions of the source cell-like immune cells, cancer cells or erythrocytes. This allows them to evade the immune system, and even in some cases, allows homing capabilities for cancer cell targeting [92]. A study conjugated gold NPs with folate and methotrexate in breast cancer cell lines due to the high expression of folate receptors. Low-level laser therapy had a proliferative effect on the breast cancer cell line. The combination of chemo and PTT with the functionalized NPs shows a significantly higher appetitive effect due to their targeting ability [93]. Table 1 below shows the gold NPs that have been investigated for drug delivery [94].

Breast cancer is often diagnosed with molecular imaging, and an NP conjugate with targeting moiety significantly enhances the output of optical imaging and can be used as a carrier in chemotherapy [95]. A polydopamine-coated magnetite NP and sphere with PAMAM dendrimers that were functionalized with NHS-PEG-Mal(N-hydroxysuccinimide-polyethylene glycol-maleimide) linker was developed to be able to functionalize with a folic acid derivative, which is a targeting moiety that can effectively kill cancer cells in dual chemo and PTT in liver cancer [96]. Microbubbles, when stabilized by a coating of magnetic or drug-containing NPs, have useful usages in theranostic applications. These microbubbles allow the transport of more efficient NP-mediated drug delivery [97]. Graphene-based NPs show good potential in photo chemotherapy. A study synthesized reduced graphene-based NPs with excellent biocompatibility capable of loading anticancer drugs for photo chemotherapy [98]. A study synthesized the polymerization of 3-caprolactone, 1,4,8-trioxa [4.6]spiro-9-undecanone and poly NPs for bladder cancer to be used as a chemotherapeutic agent with loaded DOX and zinc phthalocyanine, which enables synergistic PDT [99]. The potential risk of using NPs has yet to be fully explored. They pose a risk that is beyond the scope of chemical drug delivery. They can cross barriers that are not accessible to many other particles such as crossing the blood–brain barrier [9].

## 7. Clinical Research

Functionalized nanocarriers based on nanoparticles have been developed to improve the therapeutic efficiency of chemotherapy combined with other treatment options. The advantages of functionalized nanocarriers, namely, passive targeting capacity by the enhanced permeation and retention, ability to load drugs for targeting modification and the large surface-to-volume ratio, made various clinical research studies focusing on combined therapy possible [100]. For example, Katragadda et al. [101] demonstrated a safe and efficacious nanosized formulation for the delivery of paclitaxel and 17-AAG combination therapy, which has shown meagre responses in phase 1 clinical trials. Liu et al. [102] developed novel nanoparticles based on polymeric microspheres loaded with two anticancer drugs for pulmonary delivery. The in vivo pharmacokinetic and biodistribution studies showed that the microspheres demonstrated a prolonged circulation time and could accumulate in the lung. Araujo et al. [103] summarized the tyrosine kinase inhibitors in clinical practice for solid tumour treatment (Table 2). As SRC is a tyrosine kinase important in the oncogenic and bone-metastatic processes, it is a potential therapeutic agent to treat solid tumours. Dasatinib is one of the SRC inhibitors now being developed and is the most studied inhibitor. The current results provide valuable information to investigate if targeting SRC exhibits a viable therapeutic strategy. To date, various carrier-free prodrug NPs based on dasatinib have been designed. In vivo and in vitro experiments showed that the NPs had excellent antitumour activity and reduced toxicities [104].

## 8. Future Prospects

In this topical review, though cancer nanotheranostics is quite a novel field within these last 10 years, it has high potential to be applied extensively for cancer therapy in personalized medicine oncology. From the current works and results, it can be seen that more efforts should be taken to study the microbiological environment of the disease, and investigate the stimuli-responsive nanomedicines and co-delivery of drugs using nanocarriers. Moreover, further work should focus on the development of a novel preclinical model resulting in the potential for more accurate clinical predictability. This should lead to more clinical trials on nanotheranostics. Regarding nanomaterials, future work should focus on the design and synthesis of functionalized nanoparticles in active delivery systems, and in targeted tumour and cancer marker detection in the human body serum.

## 9. Conclusions

Cancer treatment has advanced significantly over the last 10 years and it continues to advance. The development of more functionalized nanoparticles allows cancer therapy to be more precise and imaging modalities to provide more enhances images. The combination of imaging modalities and therapeutic application allows for more accurate patient-specific treatment and it is complemented by a new theranostic nanoagent, which can serve multiple purposes in combination modalities. This review is particularly important for researchers in either cancer diagnosis or therapy to see the big picture of the recent advances in nanotheranostics. Through understanding the current development and progress of functionalized nanoparticle application in theranostics, they can find out the most promising study directions in the future.

## Figures and Tables

**Table 1:** Application of gold NPs in drug delivery. Reproduced with permission from [94].

Nanoparticle	Nanoparticle Size (nm)	Outcome	Cell Lines
MTX-AuNP	8–80	Higher cytotoxicity towards numerous cell lines as compared to free MTX. Suppression of tumour growth with MTX-AuNP but not with free MTX.	Lewis lung carcinoma (LL2) cells
DOX-Hyd@AuNP	30	Enhanced toxicity against multi drug-resistant cancer cells.	MCF-7/ADR cancer cells
(Pt(R,R-dach))-AuNP	26.7	Platinum-tethering exhibited higher cytotoxicity as compared to free oxaliplatin that could enter the nucleus.	A549 lung epithelial cancer cell line, HCT116, HCT15, HT29 and RKO colon cancer cell lines
Tfpep-AuNP conjugated with photodynamic pro-drug Pc 4	5.1	Cellular uptake of targeted particles was significantly higher than that of the non-targeted ones.	LN229 and U87 human glioma cancer lines
CPP-DOX-AuNP	25	Higher cell death as compared to previously tested 41 nm AuNP.	HeLa cells and A549 cells
FA-Au-SMCC-DOX		Enhanced drug accumulation and retention as compared to free DOX in multi drug-resistant cancer cells.	HepG2-R, C0045C and HDF
FA-BHC-AuNP	20–60	Increased efficacy of BHC against cancer cells.	Vero and HeLa
Au-P(LA-DOX)-b-PEG-OH/FA NP	34	Enhanced cellular uptake and cytotoxicity against cancer cells.	4T1 mouse mammary carcinoma cell line
DOX@PVP-AuNP	12	Induction of early and late apoptosis in lung cancer cells and upregulation of tumour suppression genes.	A549, H460 and H520 human lung cancer cells
DOX-BLM-PEG-AuNP	10	Enhanced half-maximal effective drug concentration, providing rationale for chemotherapy using two drugs.	HeLa cells
EpCam-RPAuN	48	The biomimetic nanoparticle loaded with PTX was used in combination treatment (PTT and chemotherapy).	4T1 mouse mammary carcinoma cell line

AuNP: gold nanoparticle, AuN: gold nanocage, BHC: berberine hydrochloride, BLM: bleomycin, CPP: cell penetrating peptides, DOX: doxorubicin, EpCam: epithelial cell adhesion molecule, FA: folic acid, Hyd: hydrazone, MTX: methotrexate, PEG: poly ethylene glycol, PLA: poly L-aspartate, (Pt (R,R-dach)): active ingredient of oxaliplatin, PTT: photothermal therapy, PTX: paclitaxel, PVP: polyvinylpyrrolidone, SMCC: succinimidyl 4-(N-maleimidomethyl) cyclohexane-1-carboxylate, Tfpep: transferrin peptide.

**Table 2:** Some tyrosine kinase inhibitors used in clinical practice. Reproduced with permission from [103]. Copyright 2010 Elsevier.

Tyrosine Kinase Inhibitor	Kinase Target(s)	FDA-Approved Indications
Dasatinib (Sprycel)	SRC, SFKs, BCR-ABL, c-KIT, PDGFR, c-FMS, EPHA2	CML (2nd-line), Ph + ALL
Erlotinib (Tarceva)	EGFR	NSCLC
Gefitinib (Iressa)	EGFR	NSCLC
Imatinib (Gleevec/Glivec)	BCR-ABL, c-KIT, PDGFR	CML, Ph + ALL, GIST
Lapatinib (Tykerb)	EGFR, HER2/neu	Advanced breast cancer
Nilotinib (Tasigna)	BCR-ABL, c-KIT, PDGFR	CML (2nd-line)
Sorafenib (Nexavar)	VEGFR, PDGFR	Renal cell carcinoma, hepatocellular carcinoma
Sunitinib (Sutent)	VEGFR2, PDGFR, c-KIT, FLT3	GIST, renal cell carcinoma

CML, chronic myeloid leukemia; EGFR, epidermal growth factor receptor; EPHA, ephrin A; FLT3, FMS-like tyrosine kinase 3; GIST, gastrointestinal stromal tumours; NSCLC, non-small cell lung carcinoma; PDGFR, platelet-derived growth factor receptor; Ph + ALL, Philadelphia chromosome–positive acute lymphoblastic leukemia; VEGFR2, vascular endothelial growth factor receptor-2.

## Data Availability

No new data were created or analyzed in this study. Data sharing is not applicable to this article.
